# Monolayer optical memory cells based on artificial trap-mediated charge storage and release

**DOI:** 10.1038/ncomms14734

**Published:** 2017-03-24

**Authors:** Juwon Lee, Sangyeon Pak, Young-Woo Lee, Yuljae Cho, John Hong, Paul Giraud, Hyeon Suk Shin, Stephen M. Morris, Jung Inn Sohn, SeungNam Cha, Jong Min Kim

**Affiliations:** 1 Department of Engineering Science, University of Oxford, Parks Road, Oxford OX1 3PJ, UK; 2 Department of Chemistry, Ulsan National Institute of Science and Technology (UNIST), UNIST-gil 50, Ulsan 44919, Republic of Korea

## Abstract

Monolayer transition metal dichalcogenides are considered to be promising candidates for flexible and transparent optoelectronics applications due to their direct bandgap and strong light-matter interactions. Although several monolayer-based photodetectors have been demonstrated, single-layered optical memory devices suitable for high-quality image sensing have received little attention. Here we report a concept for monolayer MoS_2_ optoelectronic memory devices using artificially-structured charge trap layers through the functionalization of the monolayer/dielectric interfaces, leading to localized electronic states that serve as a basis for electrically-induced charge trapping and optically-mediated charge release. Our devices exhibit excellent photo-responsive memory characteristics with a large linear dynamic range of ∼4,700 (73.4 dB) coupled with a low OFF-state current (<4 pA), and a long storage lifetime of over 10^4^ s. In addition, the multi-level detection of up to 8 optical states is successfully demonstrated. These results represent a significant step toward the development of future monolayer optoelectronic memory devices.

## Abstract (web summary)

Memory devices are key building blocks of image sensing circuitry. Here, the authors demonstrate a MoS_2_ monolayer optoelectronic memory device based on charge trapping and subsequent optically-induced charge release, capable of 12-bit operation.

The past few decades have seen a tremendous growth in the capabilities of silicon-based optoelectronic devices that convert photonic energy into electrical signals, which are crucial components for light-sensing applications. However, the continued advances made using bulk semiconductors are expected to reach a fundamental physical limitation regarding the development of new flexible, wearable, and transparent technology, particularly those that require miniaturization. Monolayer transition metal dichalcogenides (TMDCs) have recently received considerable attention due to their unique and unprecedented electrical and optical properties^1^,^2^,^3^,^4^,^5^ compared to those of their bulk counterparts. For example, MoS_2_ undergoes a transition from an indirect to direct bandgap when the thickness is reduced to a monolayer (∼0.7 nm), which is accompanied by a dramatic enhancement of the photoluminescence (PL) efficiency and strong light-matter interactions^3^,^6^,^7^ due to the quantum confinement that occurs in layered d-electron materials^8^. These attractive features of monolayer TMDC make it a promising material for novel flexible and wearable optoelectronics^9^,^10^,^11^ as well as novel electronic devices^12^,^13^,^14^,^15^. Accordingly, various monolayer MoS_2_-based photodetectors have been shown to exhibit outstanding optical properties with a high photoreponsivity^16^,^17^,^18^,^19^,^20^,^21^. However, the very large surface-to-volume ratio of the MoS_2_ monolayer makes it particularly sensitive to the surrounding environment such as defects on the underlying substrates or physisorbed gas molecules, leading to electron trap sites that occur due to local potential fluctuations^22^,^23^,^24^,^25^. Therefore, charge trapping in photodetectors can play an important role in determining both the response times and the light intensity-dependent photoresponse^12^,^13^. Recent studies on electrical memory devices^26^,^27^,^28^,^29^ have provided useful information about the modulation of the charge trapping density.

Compared with studies on photodetectors, relatively little research has been done to investigate the optoelectronic memory capability of devices using TMDC materials. Optoelectronic memory devices can provide a simpler approach in terms of device integration in image sensor circuitry compared with that of conventional image sensors containing a combination of photodetectors and electronic memory devices. Recently, several concepts for optoelectronic memory devices using an atomically thin film have been reported^30^,^31^. Along these lines, a hybrid structure with graphene on a multilayer MoS_2_ flake showed considerable photoresponsive properties^30^. However, the dark current level was extremely high at around hundreds of μA due to the high electrical conductivity of graphene and hence the ON/OFF current ratio remained <2, which makes it difficult to clearly discern currents between the dark and light conditions. Mechanically exfoliated few-layered CuIn_7_Se_11_ optoelectronic memory structures, similar to a charge-coupled device (CCD), have also been reported^31^. In this case, the retention time was relatively short on the order of 50 s and the ON/OFF ratio was found to be less than 10 as a result of the relatively low photosensitivity of the material, making it unsuitable for high-quality image sensing devices. Monolayer photonic memory devices with acceptable performance for practical image sensing applications have, to our knowledge, not yet been demonstrated.

Here we show a new class of MoS_2_ monolayer optoelectronic memory devices based on charge trapping at the MoS_2_/SiO_2_ dielectric interface along with the subsequent optically-induced charge release, which allows for not only a high ON/OFF ratio with a linear response to the optical dose, but also a long retention time.

## Results

### Charge trapping on monolayer MoS_2_

**Figure 1:**
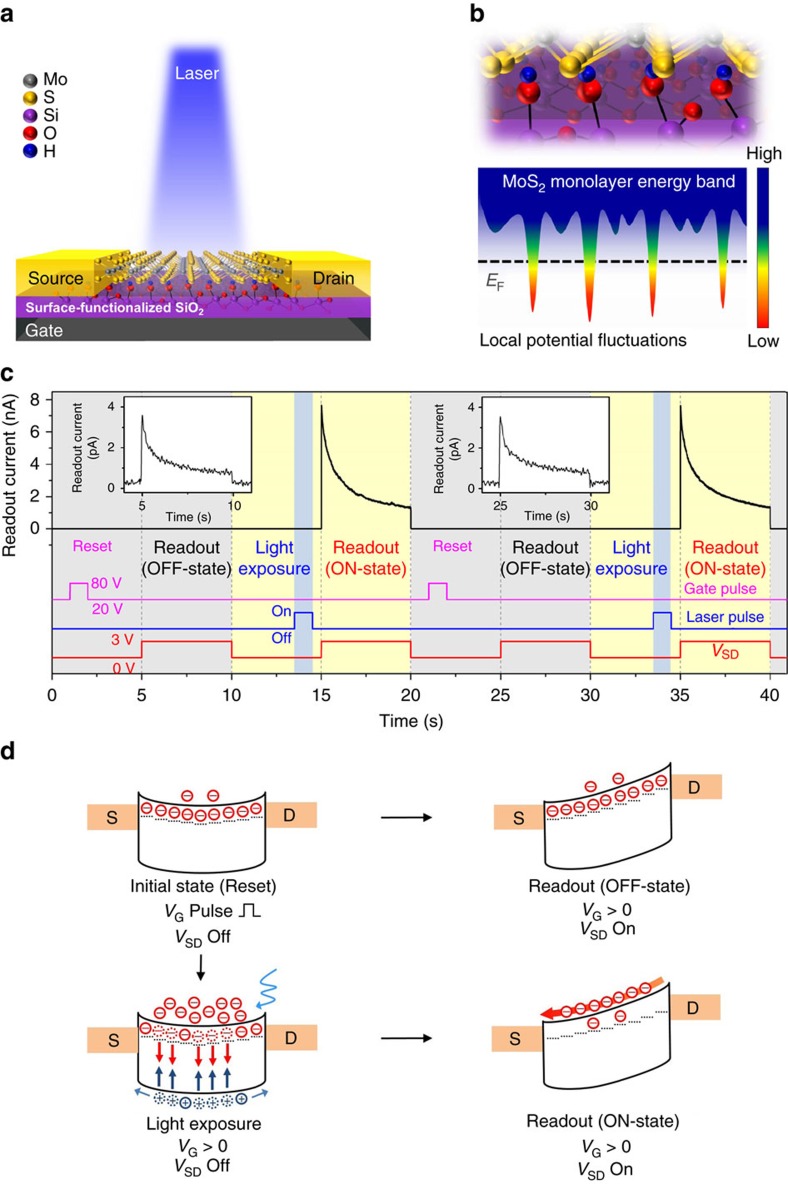
Optoelectronic memory structure and basic functions. (**a**) Schematic of the fabricated memory device where the MoS_2_/SiO_2_ interface is functionalized with silanol groups. (**b**) Schematic of the enlarged MoS_2_ atomic structure with the functional groups and a simplified energy band structure showing the local potential fluctuations induced by intentionally generated artificial trap sites. The colour bar indicates the relative electron energy level. (**c**) Operating sequence and readout current obtained from the optoelectronic device. The blue vertical shaded column indicates the period of time when the device was illuminated with a 450 nm laser with a power of 19 nW (*P*_LED_). A gate pulse (*V*_G_=80 V) and a source-drain readout bias (*V*_SD_=3 V) were applied for the reset and readout operations, respectively. (**d**) Energy band diagram of the optoelectronic device for the basic operations, depicting the initial state (reset operation), light exposure, and readout for ON- and OFF-states. The dotted lines under the conduction band represent the artificially introduced electron trap sites at the interface between the channel and the underlying SiO_2_ substrate.

Figure 1a shows the schematic of the proposed optoelectronic memory device based on a field-effect transistor (FET) structure. Large-scale monolayer MoS_2_ with single crystalline structures of around 100 μm was synthesized by vapour phase transport coupled with a surface functionalization process (see Methods section and Supplementary Figs 1–3)^32^,^33^,^34^. Because of the high surface-to-volume ratio, charge trapping originating from defects at the inherently imperfect MoS_2_/SiO_2_ interface is an inevitable phenomenon that can cause electrical hysteresis^22^,^35^. Thus, to rule out any unintended defect issues and successfully exploit the charge trapping characteristics at the interface, oxygen plasma treatment was intensively carried out on the SiO_2_ substrates before the growth of the monolayer. Note that such treatment not only removes any contaminants that act as interfacial defect sites, but also induces a chemically and structurally tailored surface layer on the SiO_2_ with functional silanol groups (Si-OH) that exhibit strong polar interaction^24^,^36^,^37^, hence causing a direct modulation of the energy band structure in monolayer MoS_2_ (Supplementary Figs 4 and 5). Explicitly, this results in uniformly distributed local potential fluctuations that are able to trap electrons energetically^24^,^37^ (Fig. 1b). On the basis of the hysteretic behaviour in the electrical transport^22^,^26^ (Supplementary Fig. 6 and Supplementary Note 1) as well as a more intense and blue-shifted PL spectra^4^ with an increase in the treatment time (Supplementary Fig. 7), it was clearly confirmed that after treating the surface for more than 5 min, charge trapping at artificially generated trap sites leads to a complete depletion of the electrons from the channel when sweeping the gate voltage back from a large gate voltage above 60 V to below 20 V. In contrast, the devices on a pristine substrate without the surface functionalization were found to exhibit weaker charge trapping and depletion characteristics. In addition, it was observed that the photoresponsivity of the surface-treated device with a basic phototransistor structure was still high enough to be used as an optical memory cell, even though the photoresponsivity decreases slightly compared to that of the device on the pristine substrate (Supplementary Fig. 8).

### Optical memory operation mechanism

Figure 1c,d show the experimental results for the basic memory functions and the operating mechanism of our optical memory cell, respectively. Since the MoS_2_ channel exhibits n-type properties, a positive gate voltage (*V*_G_=20 V) is applied without a source-drain bias to form the potential well with a bending of the energy band between two electrodes that act as Schottky barriers. When a 1 s gate pulse (80 V) is applied (Reset operation), a considerable number of electrons in the channel will be filled into the artificial trap sites due to the increase in the Fermi level^38^. Therefore, as the applied gate voltage returns to 20 V, the electron concentration in the conduction band will dramatically decrease (Initial state) because of the large number of trapped electrons, which can screen and attenuate the gate electric field to the channel (gate screening effect)^26^,^39^. Consequently, when a source-drain bias (*V*_SD_=3 V) was applied without illumination (Readout for OFF-state), the current level dropped to below 4 pA, and the readout charge integrated for 1 s was found to be less than 2 pC (left inset of Fig. 1c). Note that before the reset operation, a much larger current can flow with the application of a source-drain bias on the order of tens of nAs as shown in Supplementary Fig. 9. On the other hand, to address the optical memory storage capability, the device channel is illuminated with a 450 nm laser pulse for 1 s (Light exposure), which, in turn, generates electron-hole pairs in the MoS_2_ monolayer. Photo-generated holes can easily escape from the channel through a bending upwards of the energy band and these can also release trapped electrons through surface electron-hole recombination^17^, thus leaving behind the accumulation of photo-generated electrons in the conduction band. In addition, the removal of trapped electrons on the MoS_2_/SiO_2_ interface alleviates the gate screening effect, resulting in more electrons in the conduction band. As a result, substantially more electrons can be stored in the potential well with a longer lifetime. During the waiting time after the removal of the light source, the device is kept in dark conditions under the same gate voltage, but still without a source-drain bias voltage. Finally, when the readout bias was applied to extract the accumulated charge carriers from the potential well after a duration of 500 ms (Readout for ON-state), the readout current was found to increase to 7.7 nA. Because the current level has a tendency to return to the original current level that normally flows through the channel at the applied gate and source-drain bias by releasing the additionally stored charge, some relaxation was observed. In this case, the integrated readout charge was found to reach 4.6 nC. With these values, the ON/OFF ratio of the readout charge was calculated to be around 3,100, which enables us to effectively detect and discern a wide range of exposure dosage. It is important to note that a similar amount of readout charge was obtained from ten other devices under the same light exposure conditions, which demonstrates the reproducible device performance (Supplementary Fig. 10). On the other hand, the ON/OFF ratio for the readout charge was found to be significantly reduced to below 2 for the pristine substrate, which is consistent with previously reported devices^31^, because the relatively high readout current obtained for the OFF-state could not be easily suppressed without the artificial trap sites (Supplementary Fig. 11). We further demonstrated that the device could be operated sequentially using the reset operation after each readout for the ON-state (right inset of Fig. 1c). This operating principle can give our device a critical advantage regarding optoelectronic memory, as discussed later in this article.

### Linearity of the optical memory device

**Figure 2:**
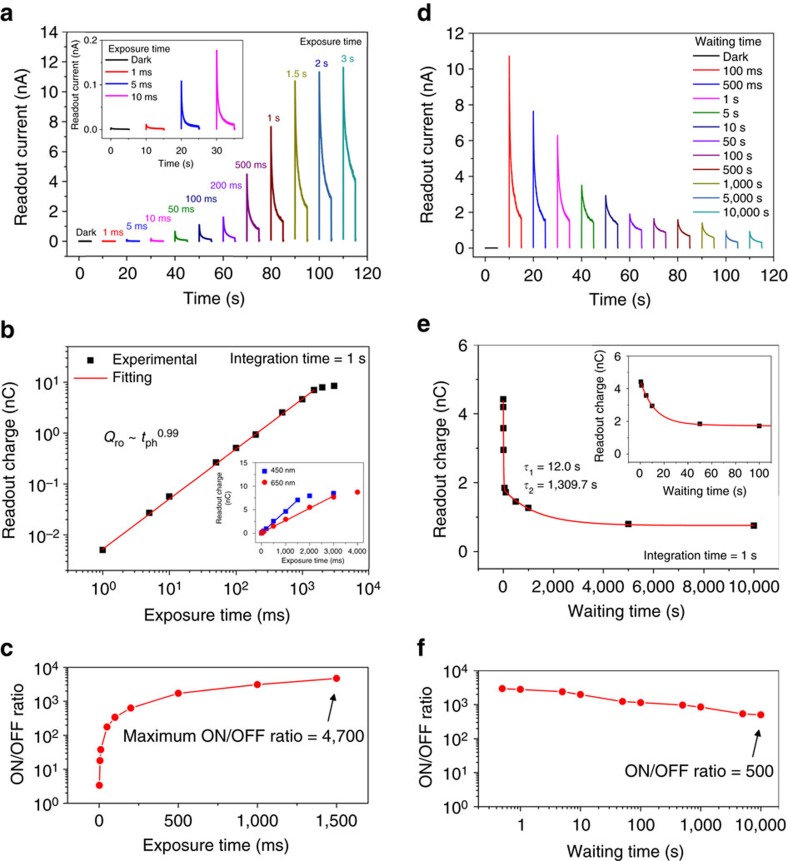
Photoresponse characteristics for different exposure and waiting times. (**a**) Readout current as a function of light exposure times up to 3,000 ms. The plots were separated with an interval of 10 s, regardless of the time that was measured initially. The readout bias voltage was applied after a 500 ms waiting time. The inset represents an enlarged view of the readout current for different exposure times from dark conditions up to 10 ms. (**b**) The extracted readout charge (in log scale) obtained by integrating the readout current for 1 s with increasing exposure time. The fitted line (red), using the power-law function, indicates that the readout charge is linearly proportional to the exposure time or dose for the range below 1,500 ms. The inset shows the readout charge (in linear scale) as a function of exposure times for a different excitation wavelength (*λ*=650 nm) showing a similar linear dependence and almost the same saturated charge value. (**c**) The ON/OFF readout charge ratio as a function of exposure times. The LDR was determined to be 4,700 (73.5 dB) at a 1,500 ms exposure time. (**d**) Readout current in the ON-state as a function of waiting times up to 10^4^ s. The graphs were spread separately with an interval of 10 s, regardless of the time measured initially. (**e**) The extracted readout charge obtained by integrating the readout current for 1 s with increasing waiting time. Two decay time constants, 12.0 and 1,309.7 s, were estimated by using the double exponential fitted line (red). The enlarged graph of the readout charge extracted for a waiting time up to 100 s is shown in the inset. (**f**) The ON/OFF ratio for different waiting times. The ON/OFF ratio was found to be maintained above 500 even after waiting for 10^4^ s.

One of the most important key parameters for practical applications, especially image sensing, is a linear responsivity with respect to the light exposure dosage so as to be able to detect the contrast of images accurately. If the photo-response deviates from linearity or saturates for a small amount of exposure, it is then difficult to quantify the exact dosage. Figure 2a shows the readout current measured after illumination as a function of light exposure times from 1 to 3,000 ms. Photo-responsive memory features were observed even in a short light exposure time of 1 ms, corresponding to an optical energy smaller than 17 pJ (inset of Fig. 2a). The readout charge was also extracted by integrating the readout current for 1 s after a waiting time of 0.5 s without a source-drain bias voltage (Fig. 2b). The fitted result clearly shows that the readout charge (*Q*_ro_) is linearly dependent on the light exposure time (*t*_ph_) up to 1,500 ms (*Q*_ro_≈*t*_ph_^α^, α=0.99) and is directly proportional to the dosage or the number of incident photons. However, when the exposure time increased above 2,000 ms, the readout charge started to deviate from a linear relationship and then saturated at around 7.1 nC (inset of Fig. 2b). This saturated charge level was almost the same as that obtained when irradiated by a 650 nm red laser, which shows that the device has a specific full-well charge capacity regardless of the wavelength of light (inset of Fig. 2b and Supplementary Fig. 12). In addition, the collected readout charge at various integration times from 50 to 5,000 ms also depends linearly on the exposure time, which means the integration time can be selected according to the device requirement (Supplementary Fig. 13). We also confirmed that the readout charge is linearly proportional to light intensity from 19 pW to 19 nW (Supplementary Fig. 14).

On the basis of the ON/OFF ratio of the readout charge as shown in Fig. 2c, we found that the linear dynamic range (LDR), which represents the ratio of the full-well charge capacity to the dark readout charge within the linear region, was found to be 4,700 (73.4 dB) after a 1,500 ms exposure time. This LDR is larger or comparable to consumer grade Si-based CCDs (60∼70 dB)^40^, providing the possibility of multi-level detections with a 12-bit analog-digital converter. These results strongly indicate that the high sensitivity is achieved and maintained over a wide exposure timescale because the charge quantity is directly associated with the dosage.

### Charge storage time

Another important parameter for image sensing and memory devices is the charge storage lifetime. Figure 2d shows the readout current in the ON-state as a function of the waiting time before readout. The light exposure duration was fixed at 1 s, and the readout was done for 1 s, which is identical to that of the exposure time-dependent experiment described above. To examine the progressive time-evolution of electrons stored in the potential well, we plotted the integrated readout charge as shown in Fig. 2e. The resulting amount of the readout charge was adequately fitted with two exponential functions expressed as


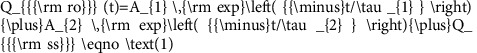


where *τ*_1_, *τ*_2_ are the time constants of the decay process, *A*_1_, *A*_2_ are the constants determined by the light intensity and other device parameters, and *Q*_ss_ is the readout charge at steady-state. The two characteristic decay time constants were estimated to be 12.0 and 1,309.7 s. These results clearly indicate that there are two different types of decay process occurring in the device. The first decay process predominantly arises from the photo-generated electrons that have an extended lifetime through the surface recombination of the photo-generated holes with the electrons trapped at the interface. This effect mainly lasts up until 100 s and then disappears completely after that time. It is believed that the photo-generated electrons stored in the potential well might escape through the electrodes during the first decay period, overcoming the Schottky barrier due to thermal fluctuations^31^,^41^.

The subsequent decay process that has an extremely slow time constant comes from the weakened gate screening effect. In other words, the original gate bias is slightly retrieved on the removal of trapped electrons at the interface through the recombination with the photo-generated holes, helping not only to maximize the induction of electrons but also to store more electrons in the enhanced potential well as a result of the larger gate field effect. It is expected that such a constructive gate field effect will last for a long time because it is not easy to return the channel to the reset state at a gate voltage of 20 V without the application of the reset gate pulse as discussed earlier (Fig. 1c), even though a small amount of electrons might be recaptured at trap sites during the waiting time. Consequently, the information can be stored and retrieved while maintaining a high ON/OFF ratio greater than 100 even after waiting for 10^4^ s, consuming ultra-low power without a source-drain bias (Fig. 2f). These results indicate that our optical memory device with a long-term storage lifetime could have, fundamentally, great potential for the development of much simpler image sensing chip architecture without the additional non-volatile memory arrays required for charge storage.

### Stability of dark current and the effects of gate voltage

**Figure 3:**
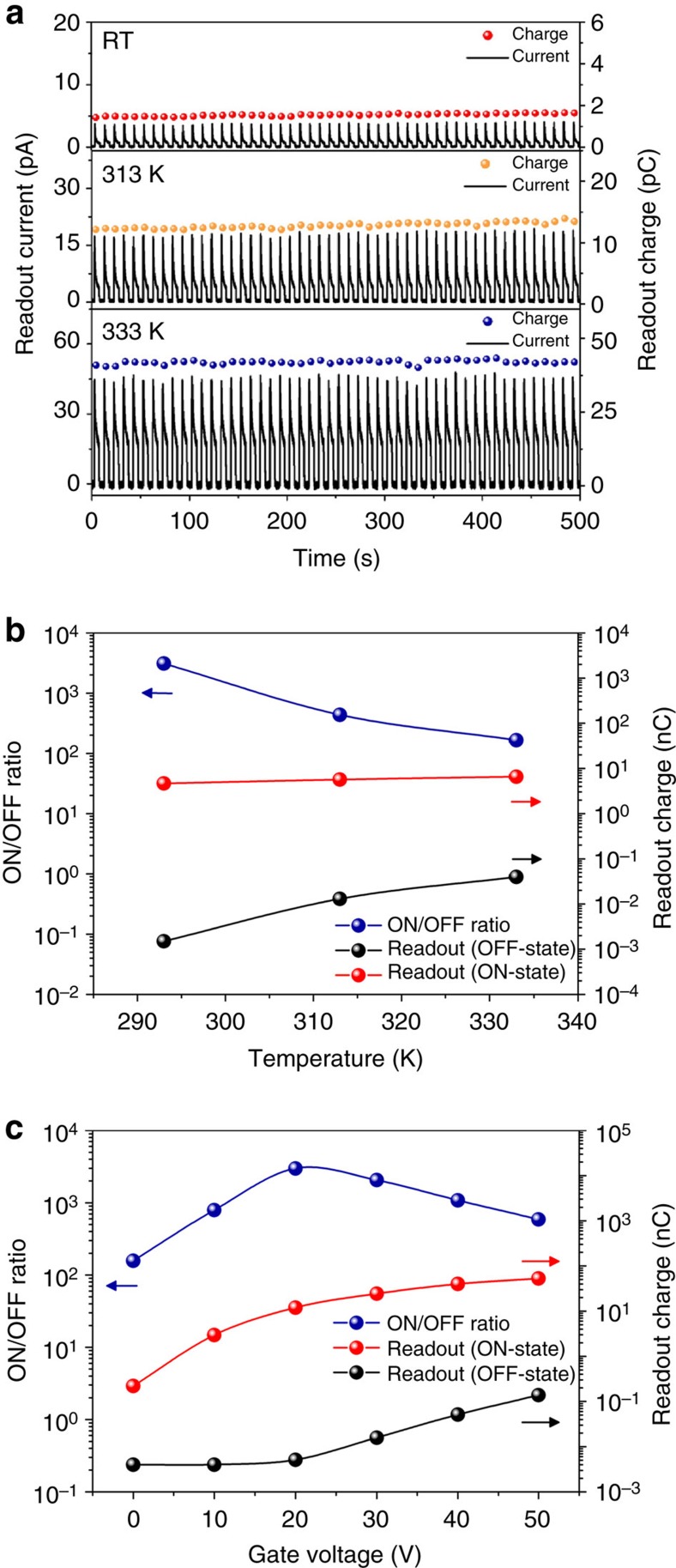
Dark readout stability and dependence on temperature and gate voltage. (**a**) Dark readout operations for 500 s at *T*=293 K, *T*=313 K, and *T*=333 K. Dark readout current was stable at three different temperatures, however, it gradually increased at higher temperatures because of electrons thermally excited from the trap sites. (**b**) Readout charge for ON/OFF-states and ON/OFF ratio as a function of temperature. (**c**) Readout charge for the ON/OFF-states and ON/OFF ratio as a function of gate voltage. The readout charge ON/OFF ratio was found to be the highest at a gate voltage of 20 V.

Since the low dark current is essential to secure a high ON/OFF ratio in optical devices, we further investigated the stability of the dark (OFF-state) readout current in our devices. Even in the absence of light exposure, thermally excited electrons can be collected in the potential well and contribute to the readout signal, decreasing the device performance. In Fig. 3a, the temperature-dependent dark readout was performed for 500 s with a period of 10 s at various temperatures ranging from *T*=293 K to *T*=333 K. When the device was kept at near room temperature (*T*=293 K), the peak of the dark current showed no significant change at about 4 pA for 500 s. Similarly, the readout charge integrated from the dark current for 1 s also maintained almost the same value of less than 2 pC for the whole process time. When the operation was carried out at *T*=313 K, the peak of the current and the readout charge increased to 17 pA and 13 pC, respectively, however, the peak of the dark current did remain stable. This trend was also observed at an even higher temperature of *T*=333 K, where we recorded a stable peak current (50 pA) and readout charge (40 pC). These results indicate that the dark current does remain stable at the given temperature. The increase of the dark current with temperature is believed to originate from electrons released from trap sites by thermal excitation, similar to the phenomena reported in CCD image sensors^42^. This thermal noise can cause the dark current to rise significantly, which directly leads to the deterioration of the readout ON/OFF ratio from 2,340 at *T*=293 K to 161 at *T*=333 K (Fig. 3b).

To address how much the initial gate bias can affect the readout current, we also investigated the device characteristics as a function of initial gate voltages under the same conditions as shown in Fig. 1c. Figure 3c shows that in the range of initial voltages from 0 to 20 V the dark readout charge maintained almost the same value at around 1.5 pC, while it increased gradually above 20 V and reached a value of 27.3 pC at 50 V, causing a significant decrease in the ON/OFF ratio. This is primarily due to the increase in the channel carrier density induced by a high gate voltage. On the other hand, the accumulated charge from the light increased rapidly from 0 to 20 V and then showed a saturated response above 20 V. These results show that only a gate voltage more than 20 V can guarantee a robust potential well to store the electrons sufficiently. Consequently, the ON/OFF ratio reached its highest value at around 3,100 when we operated the device with a gate voltage of 20 V, but it dropped sharply to 590 at 50 V, and to 160 at 0 V. From these results it is confirmed that a gate voltage of around 20 V is optimal for operating the memory cells.

### Multi-bit operations

**Figure 4:**
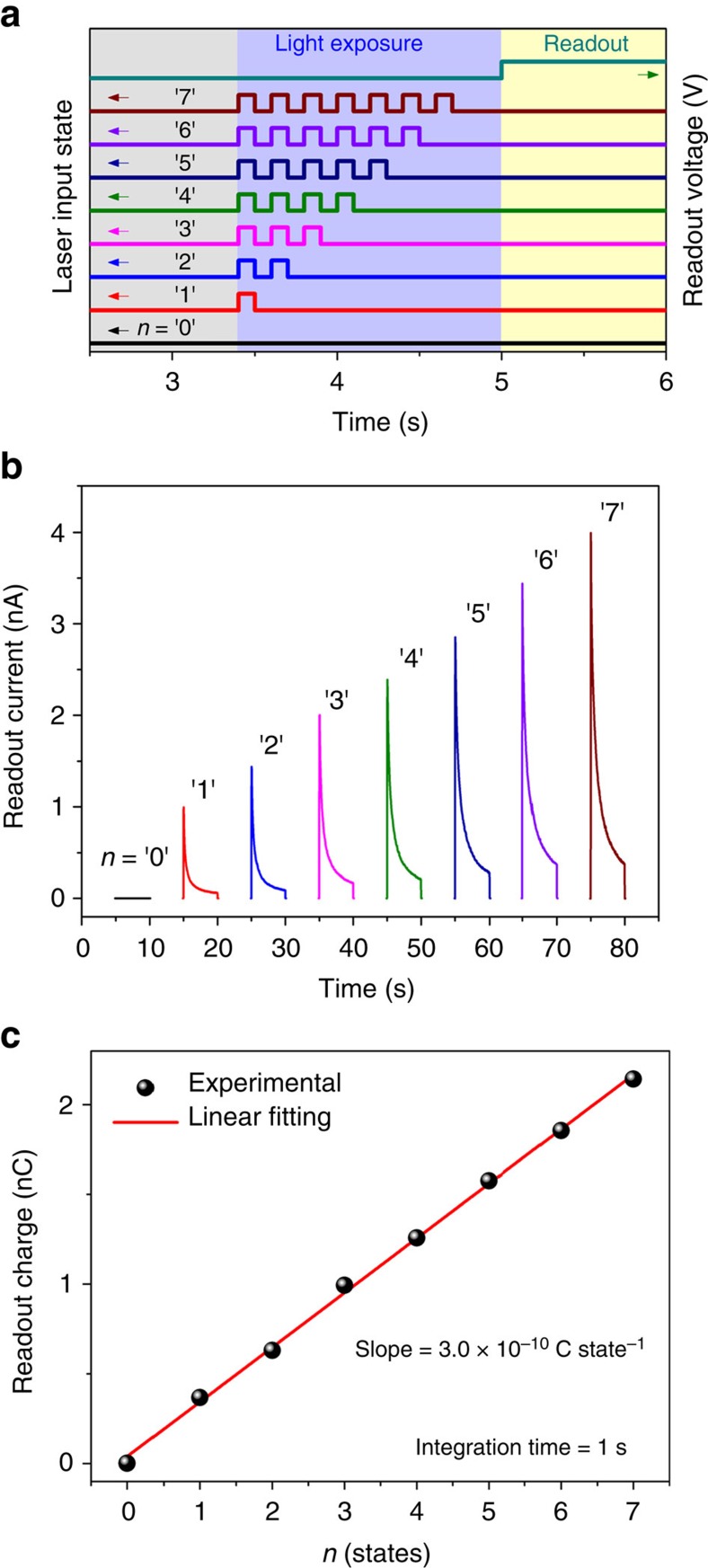
Multi-bit response of the MoS_2_ optoelectronic memory device. (**a**) The input of optical laser pulses (blue shade) and the readout stage (yellow shade). Each optical state was written into the MoS_2_ device with a 100 ms duration, and the interval between bits was also 100 ms. (**b**) Readout current as a function of the number of applied laser pulses, leading to eight different optical states (three bits). The plots were separated with an interval of 10 s, regardless of the time that was measured initially. (**c**) The collected readout charge in the ON-state recorded for 1 s. The line of best fit (red) indicates that the charge increases by 300 pC for each optical laser pulse.

The attractive optoelectronic features of our devices—large LDR, low dark current, and relatively long retention time—could potentially open up avenues for other optoelectronic applications. Figure 4 shows the multi-bit optical data storage capacity of a single MoS_2_ memory cell exhibiting eight different states defined as ‘0', ‘1' and so on. Each optical state was written into the MoS_2_ monolayer channel with a 100 ms laser pulse, and the time gap between each laser pulse was also 100 ms (Fig. 4a). As the number of applied laser pulses increased, the ON-state readout current also gradually increased (Fig. 4b), and the integrated readout charge had a linear relationship with the number of applied laser pulses as shown in Fig. 4c. The readout charge consistently increased by around 300 pC for each applied laser pulse. It was also confirmed that the multi-bit detection was reliable and reproducible with repeated switching (Supplementary Fig. 15 and Supplementary Table 1). In addition, if we could minimize the noise level of the states further, it would make it possible to implement many more states^43^. We further note that, even though multi-bit operation could be easily realized by using a single optical pulse with different light intensities during the same exposure time as shown in Supplementary Fig. 14, we have instead demonstrated multi-bit operation by controlling the number of optical pulses at the same intensity. This is because it is much more practical to use a single-intensity light source so as to simplify the system configuration and ensure cost saving, without the need for any additional components to control the light intensity. Although it still remains a challenging task for our device to exhibit permanent optical storage, these findings suggest that it has the potential for optoelectronic multi-level modulation.

## Discussion

We have proposed and demonstrated an optical memory device based on single-layered MoS_2_ through a combination of artificial trap-mediated charge trapping into local potential fluctuations and optically-induced charge generation. By optimizing the potential well structure through a modification of the interface using functional groups that are capable of localizing charge carriers, we showed that our optical device structure could controllably trap and release electric charges that were stored in trap states, allowing programmable functions for optical memory devices. The OFF-state readout current was found to be almost on the order of pA while the ON/OFF ratio of the readout charge, which exhibited a linear response, was found to exceed 4,700. These combined features make it possible to detect the contrast of an image with 12-bit levels. In addition, we have demonstrated that the device could store an optical signal for over 10^4^ s without applying a source-drain bias, thereby consuming extremely low power before the readout. Furthermore, we showed that the device could store up to eight optical states whilst maintaining a linear response. Our work opens up new opportunities to increase the utilization of TMDC materials with multiple benefits for future optoelectronic applications.

## Methods

### Chemical vapour deposition growth of single-layered MoS_2_

Silicon substrates with 300 nm of SiO_2_ were used as the growth substrates. After cleaning the substrates, they were functionalized using an oxygen plasma treatment. These target substrates for growth were loaded upside down above a crucible in the 50-mm quartz tube of the chemical vapour deposition (CVD) growth system, and source substrates with an ultra-thin and homogeneous MoO_3_ precursor film prepared using diluted precursor solutions by spin-coating were placed under the target substrates. Another crucible containing 200 mg of sulfur powder (Sigma-Aldrich) was placed upstream at the edge of the tube. The CVD growth was conducted at atmospheric pressure with a continuous flow of pure argon gas, and the detailed growth recipe is: anneal at 200 °C for 20 min under 1,000 sccm flow of argon gas to remove oxygen and water in the tube, ramp to 650 °C at a rate of 16.25 °C min^−1^ with 100 sccm of argon (as the temperature of the furnace approaches 650 °C, the temperature around the sulfur powder, placed upstream at a lower temperature near the edge of the furnace, increases to above the melting point (∼115 °C), then the sulfur starts to melt), maintain at 650 °C for 5 min and cool to room temperature.

### Device fabrication and measurement

Electrode contacts were drawn by a standard photolithography and lift-off process. The Au (50 nm) source and drain electrodes were deposited by thermal evaporation. To minimize the effects of physisorbed gas molecules such as oxygen and water on the MoS_2_ surface, all measurements were performed at room temperature after thermal annealing at around 200 °C for more than 2 h in vacuum. A 450 or 650 nm laser with transistor-transistor logic (TTL) modulation was focused onto the devices. Finally, characterization of the electrical properties was carried out using a Keithley 4200-SCS Parameter Analyzer, Lecroy HD4000 high definition oscilloscopes, Stanford Research Systems SR570 current preamplifiers, and a Cascade Microtech probe station.

### Data availability

The data that support the findings of this study are available from the corresponding authors on request.

## Additional information

**How to cite this article:** Lee, J. *et al*. Monolayer optical memory cells based on artificial trap-mediated charge storage and release. *Nat. Commun.* **8**, 14734 doi: 10.1038/ncomms14734 (2017).

**Publisher's note:** Springer Nature remains neutral with regard to jurisdictional claims in published maps and institutional affiliations.

## Supplementary Material

Supplementary Information Supplementary Figures, Supplementary Table, Supplementary Note and Supplementary References
