# Supplementary material for: Monolayer optical memory cells based on artificial trap-mediated charge storage and release
Source: Nat Commun. 2017 Mar 24;8:14734. doi: 10.1038/ncomms14734 (PMC5376667; doi:10.1038/ncomms14734)
Supplement: Supplementary Information — Supplementary Figures, Supplementary Table, Supplementary Note and Supplementary References [file ncomms14734-s1.pdf]

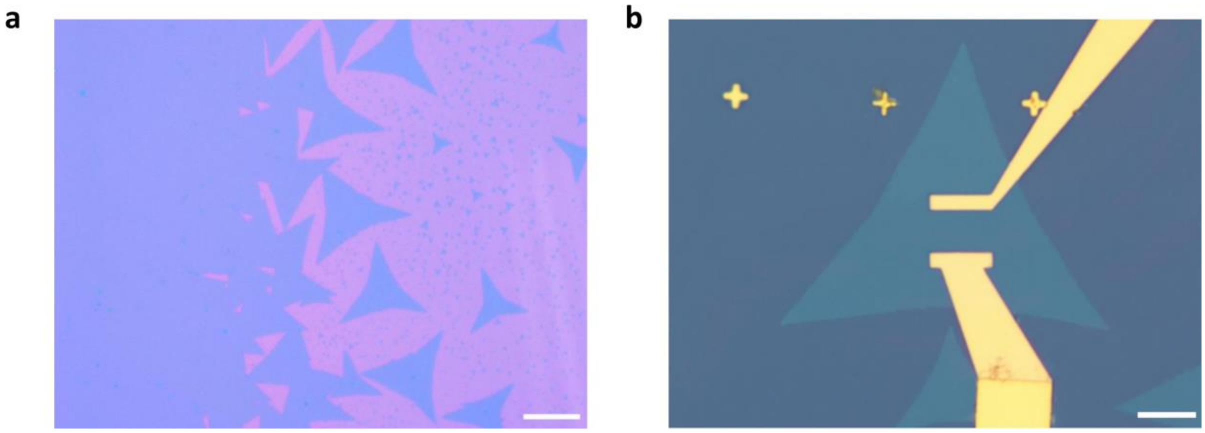

Supplementary Figure 1. Optical images of monolayer MoS_2 grown on SiO_2. a, An optical image that shows that layered MoS_2 crystals with well-defined side facets are grown on the SiO_2 substrate with a size of around 100 µm, and the crystals are merged to form a continuous layered MoS_2 film. Magenta and violet indicate the SiO_2 substrate and the MoS_2 monolayer, respectively. Scale bar: 50 µm. **b,** An optical image of the typical layered MoS_2 FET with a back-gate structure. Scale bar: 5 µm.

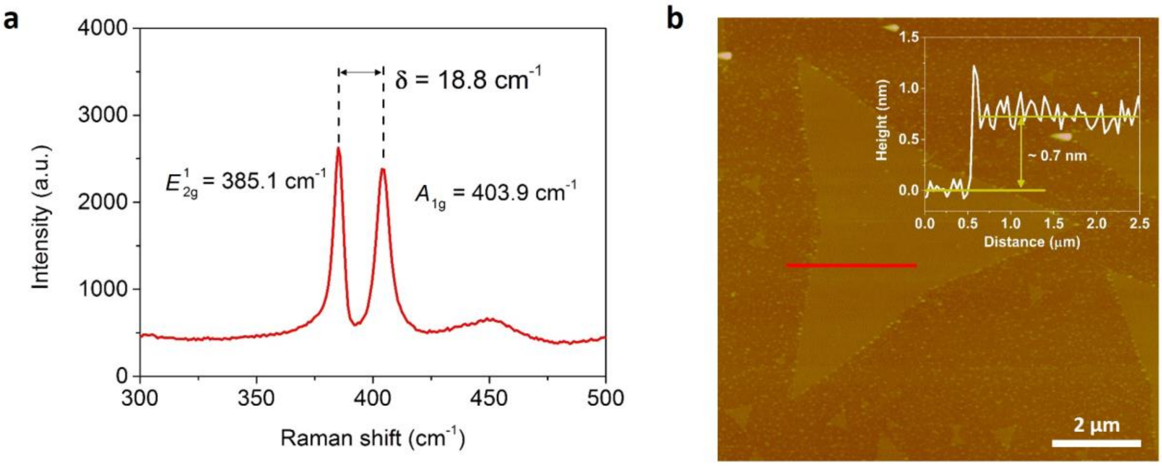

Supplementary Figure 2. Structural properties of monolayer MoS_2. a, Raman spectrum obtained from monolayer MoS_2 crystals grown on SiO_2. Measurements were obtained using a 532 nm laser. The peak distance between E^1_{2g} and A_{1g} (18.8 cm^{-1}) confirms that the as-grown MoS_2 is indeed a monolayer[1,2]. **b,** The AFM topography image of an isolated monolayer of MoS_2 and the corresponding height profile extracted from the red line (inset). The thickness of the MoS_2 layer is ~0.7 nm, determined from the height profile, providing evidence for the formation of the monolayer MoS_2.

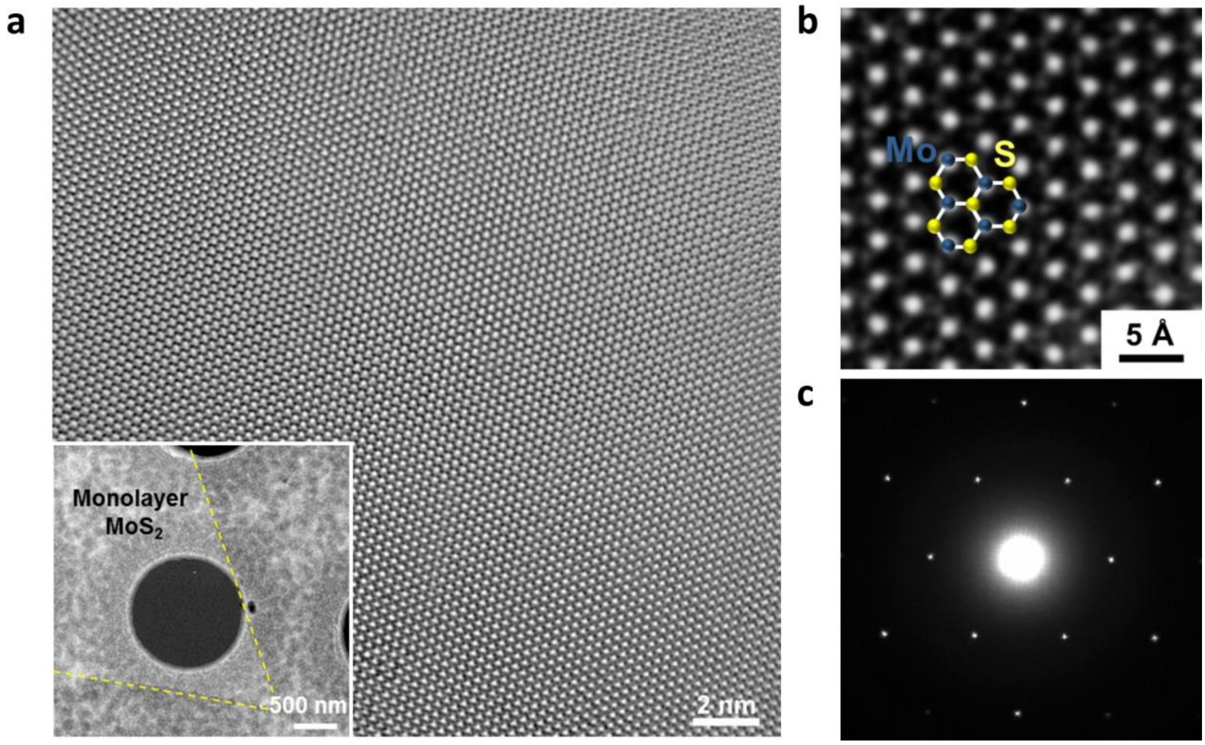

Supplementary Figure 3. Transmission electron microscopy (TEM) analysis of monolayer MoS_2. a, High-resolution TEM image of monolayer MoS_2 grown by chemical vapour deposition. Inset: bright-field TEM image of the monolayer MoS_2 flake. **b,** High angle annular dark field scanning transmission electron microscopy (HAADF-STEM) image representing the defect-free hexagonal structure of the monolayer MoS_2 flake. The bright spots are molybdenum atoms, and the grey spots are two stacked sulfur atoms. **c,** Diffraction pattern confirming the hexagonal MoS_2 structure.

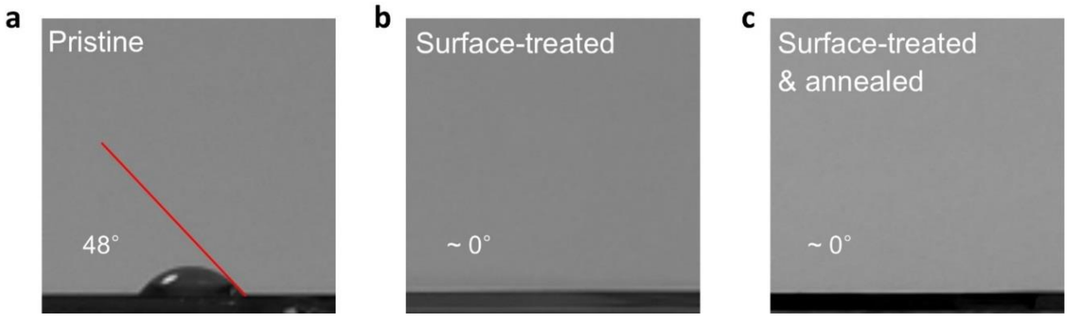

Supplementary Figure 4. Images of water contact angles on SiO_2 substrates. (a) Pristine substrate, (b) Surface-treated substrate, and (c) Surface-treated and then annealed substrate. It can be seen that the water contact angle on the pristine substrate was 48°, which means that the substrate was hydrophobic because of some organic contaminants on the surface. However, the water contact angle became almost 0° after treating the surface for 5 min, which indicates that the surface becomes hydrophilic due to the elimination of organic contaminants and the induction of Si-OH functional groups on the surface through the treatment process. Furthermore, it is observed that the surface can also maintain its hydrophilic property after annealing the substrate at the same condition with the growth process (650°C for 5 min in Ar atmosphere), but without precursors so as to rule out the possibility that the value of water contact angles on the surface-treated substrate can be affected by the precursors adsorbed on the surface.

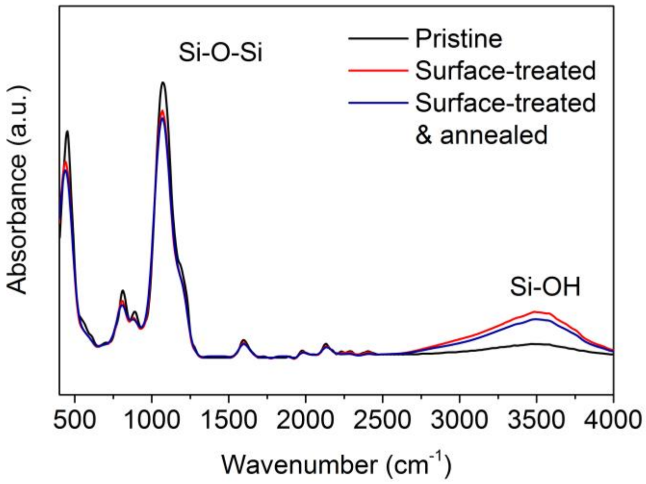

Supplementary Figure 5. FTIR analysis of the SiO_2 substrate. Before the surface treatment (black), after the treatment (red), and after annealing at 650°C following the treatment (blue). Only a negligibly small amount of –OH groups, indicated by a peak ranging from 3000 to 4000 cm^{-1}, was found before the surface treatment, while the intensity of the peak related to the Si-OH stretching mode significantly increased after the treatment. This clearly shows that the surface treatment induces Si-OH groups on the surface. In addition, similar to the water contact angle results, most of the –OH groups still remain on the surface after the annealing at 650°C for 5 min, even though the total amount of –OH groups decreases slightly.

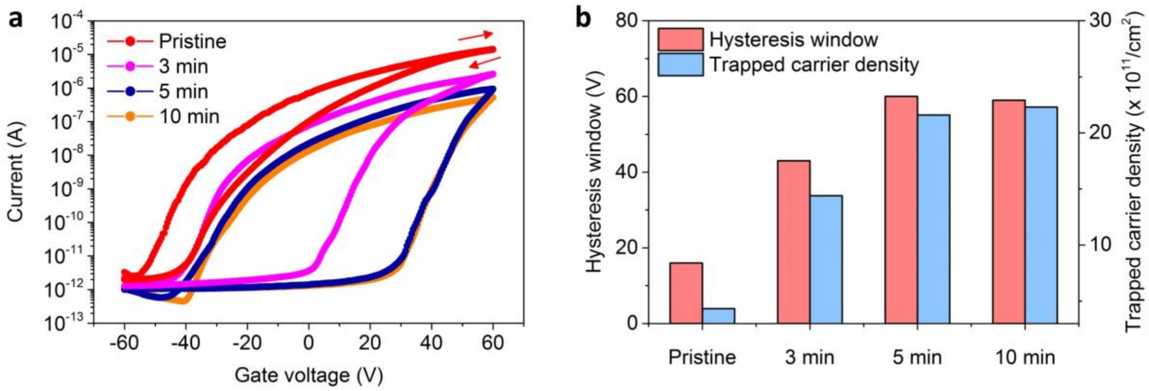

Supplementary Figure 6. Surface treatment effects on transfer curves. a, Hysteretic behaviour of the devices on the pristine substrate without surface treatment (red) and on the substrates treated for 3 min (magenta), 5 min (blue), and 10 min (orange) at V_{SD} = 3 V. The arrows indicate the gate bias sweep direction. **b,** Hysteresis windows and trapped carrier densities for the devices on the pristine substrate and on the surface-treated substrates based on the results in (a).

Supplementary Note 1. Explanation on surface treatment effects. The device on the pristine substrate without functional groups showed a mobility of 5 $cm^2\ V^{-1}\ s^{-1}$ for a voltage sweeping direction from -60 V to 60 V and an on/off ratio of ~10^7. Even though the measurement was done after thermal annealing in a vacuum, the typical hysteretic behaviour of an n-type MoS_2 FET on the pristine substrate was observed to exhibit a relatively small hysteresis window compared to devices on the surface-treated substrates as shown in Fig. S3a. The hysteresis window is defined as the maximum voltage shift at I_{SD} = 1 nA, which is related to the transition voltage in the transfer curves. It should be noted that we believe that the hysteresis observed for the monolayer MoS_2 on the pristine substrate is not significantly affected by air or oxygen, but is caused by natural defects on the underlying SiO_2 substrate. This is because the relatively small hysteresis appears to be unchanged after both thermal annealing and vacuum treatment as well as surface passivation[3,4]. On the other hand,

noticeably, the device on the surface-functionalized substrate showed a relatively reduced conductance and a larger hysteresis window of 43 V (3 min), and 60 V (5 min) due to the artificially introduced trap sites. The device on the substrate treated for 10 min showed a similar hysteretic behaviour, which means that the charge trap sites are almost saturated after treating the surface for more than 5 min. The trapped carrier density (N_{trap}) can be calculated using the parallel-plate capacitor model, with $N_{trap} = (C_{ox}\ \Delta V_{th})/e$, where C_{ox} = 11.5 nF cm^{-2}, ΔV_{th} is the difference in the threshold voltages, and e = 1.602 × 10^{-19} C is the elementary charge. The device on the pristine substrate showed ΔV_{th} = 6 V, corresponding to a trapped carrier density of 4.3 x 10^{11} cm^{-2}, while 20 V, 1.44 x 10^{12} cm^{-2} (3 min) and 30 V, 2.2 x 10^{12} cm^{-2} (5 min), and 31 V, 2.23 x 10^{12} cm^{-2} (10 min) were extracted from the device on the surface-treated substrates, respectively. These results indicate that the surface treatment induces a number of silanol groups (Si-OH) on the SiO^2 substrate so as to trap more electrons at a high positive gate voltage.

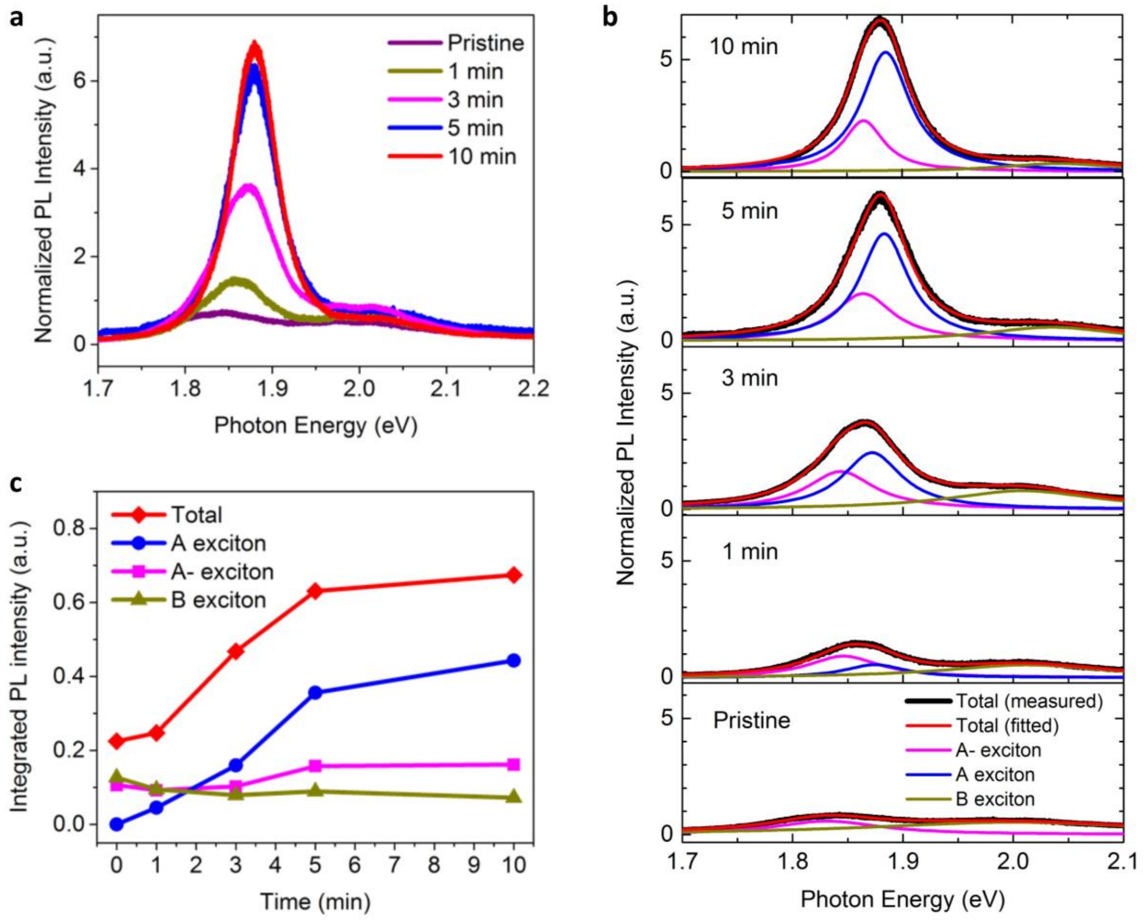

Supplementary Figure 7. Photoluminescence spectra evolution with surface treatment times. a, Normalized photoluminescence (PL) spectra obtained at each plasma treatment time (pristine, 1 min, 3 min, 5 min, and 10 min). Each PL measurement was conducted after applying a positive gate pulse for 1 s (0 V → 80 V → 0 V) in order to investigate in detail how the PL spectra behaviour is affected by the modulation in the electron density through the artificial trap sites that are induced by the surface treatment. It was clearly observed that the PL peak became more intense and shifted to blue wavelengths with an increase in the surface treatment time, although the PL peak showed a similar trend after treating the surface for more than 5 min. **b,** The analysis of PL spectra shapes was performed by deconvoluting each PL plot, which has been fitted with Lorentzian curves to calculate the intensities of the

A- exciton (trion), the A exciton, and the B exciton peaks. **c,** Integrated PL intensities of each exciton show that the intensity of the A exciton gradually increases with the plasma treatment time and then becomes almost saturated after 5 min treatment, while the intensity of the negatively charged A- exciton (trions) remains almost the same. This suggests that the A exciton recombination is dominant on the surface treated substrates due to a lowering of the Fermi energy level resulting from the induced trap states, causing a decrease in the number of excess electrons in the monolayer MoS_2. Note that it has been reported that in general, trion recombination mainly occurs for heavy electron doping[5].

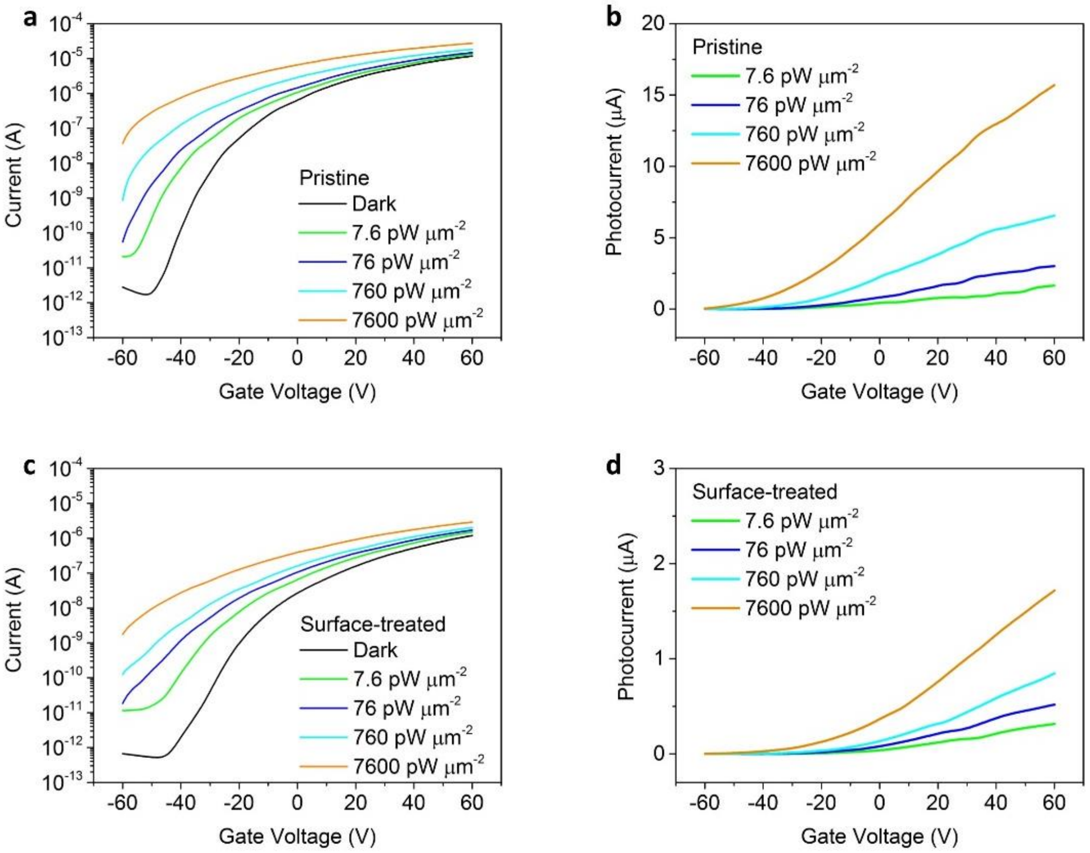

Supplementary Figure 8. **Photoresponse and photocurrent of monolayer MoS_2 photodetectors**. On pristine substrates (a, b) and surface-treated substrates (c, d). The photocurrent was obtained by subtracting the dark current from each curve in (a) and (c). The device on the pristine substrate exhibits the photoresponsivity of around 1740 A W^{-1} at a gate voltage of 60 V and an optical power per unit area of 7.6 pW cm^{-2}, which is superior or comparable to the previously reported results for phototransisotor applications[6,7]. On the other hand, the device on the surface-treated substrate shows a photoresponsivity of 415 A W^{-1} under the same condition. Even though the photoresponsivity decreases after the surface treatment, it is still much higher, compared to that of other 2D materials[8-10]. The high photoreponsivity even on the surface-treated substrate originates from the strong light-matter interaction on the monolayer MoS_2, and makes the material suitable for our memory device applications.

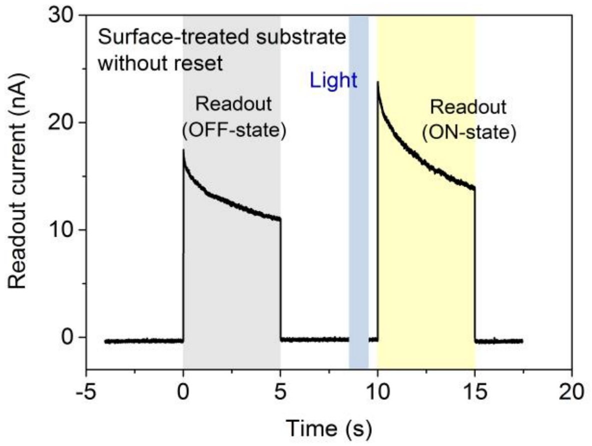

Supplementary Figure 9. Optical memory operation without reset operation. The readout current for the OFF-state was around tens of nA without the reset operation, although the readout current for the ON-state increased after light was illuminated on the device. The ON/OFF ratio of the readout charge was found to be less than 2, which made it difficult to clearly distinguish between the ON/OFF state conditions.

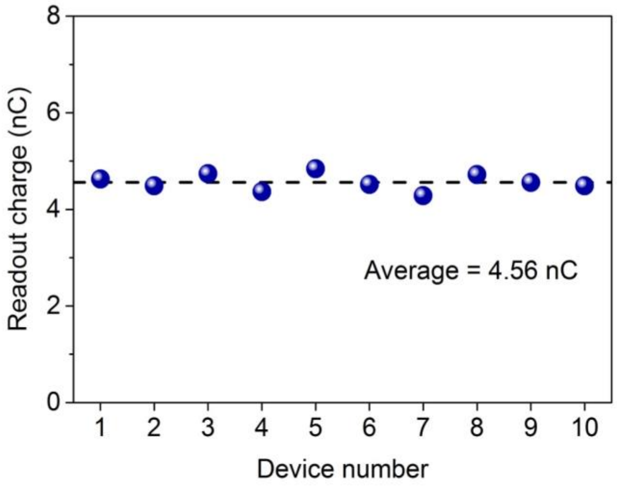

Supplementary Figure 10. Uniformity of readout charge for different memory cells. Ten different devices were tested to statistically examine the uniformity and reproducibility of the readout charge. The readout charge obtained from the ten devices was found to be within a similar range. The average value was 4.56 nC (dashed line). The light exposure time, the waiting time, and the integration time were 1 s, 0.5 s, and 1 s, respectively, for all the devices. The uniformity of the readout charge indicates that the surface treatment induces a uniform surrounding environment at the interface between the MoS_2 and the substrate.

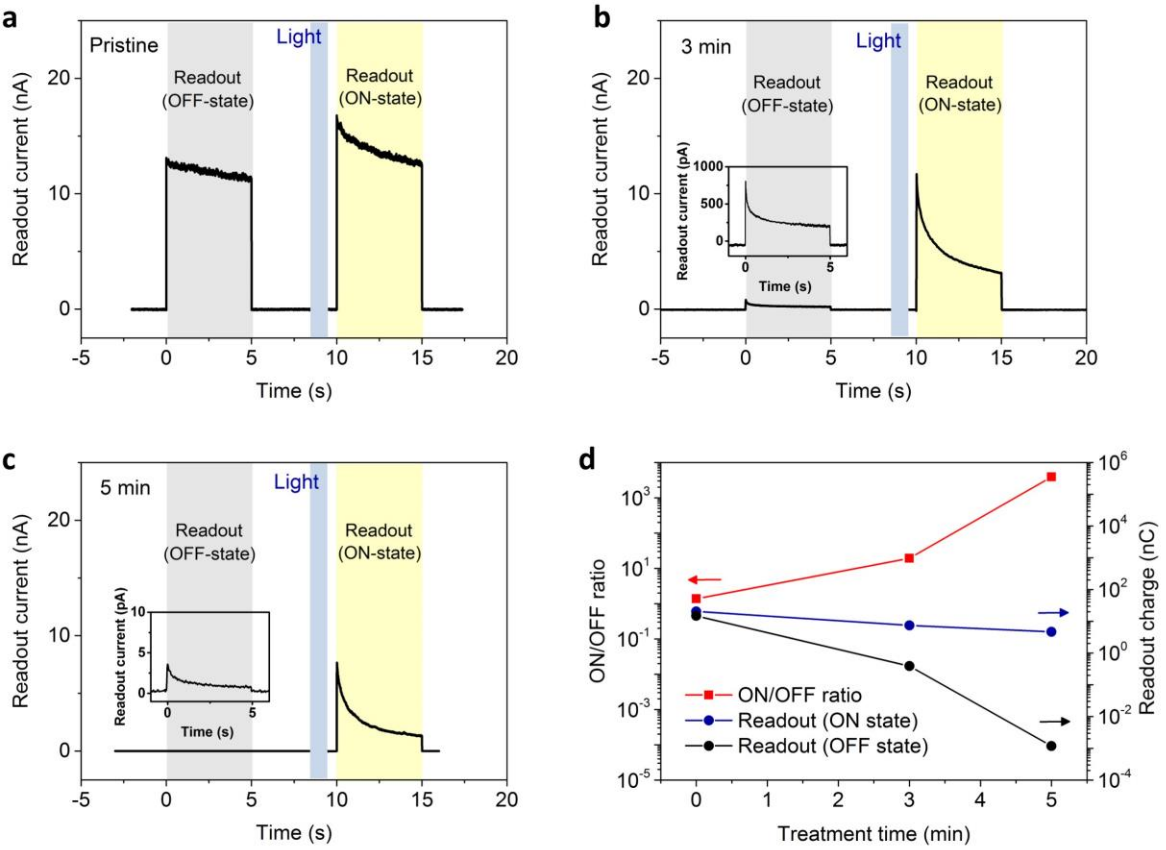

Supplementary Figure 11. Surface treatment effect on optical memory operation. a, The readout current for the OFF-state after the reset operation was found to be significantly higher for the pristine substrate. The reset effect is relatively weak because the trapped carrier density is much lower than that of the device based on the surface-treated substrate. It shows no significant difference between the OFF- and ON-state readout for a 1 s light exposure time and a 0.5 s waiting time. **b, c,** The readout current for the OFF-state dramatically decreases as the surface treatment time increases to 3 min (panel **b**) and 5 min (panel **c**) due to the artificially induced trap sites, while the readout current for the ON-state drops only slightly. **d,** As a result, the ON/OFF ratio of the readout charge sharply increases from 1.37 (for the pristine substrate) to 3930 (5 min – surface-treated substrate).

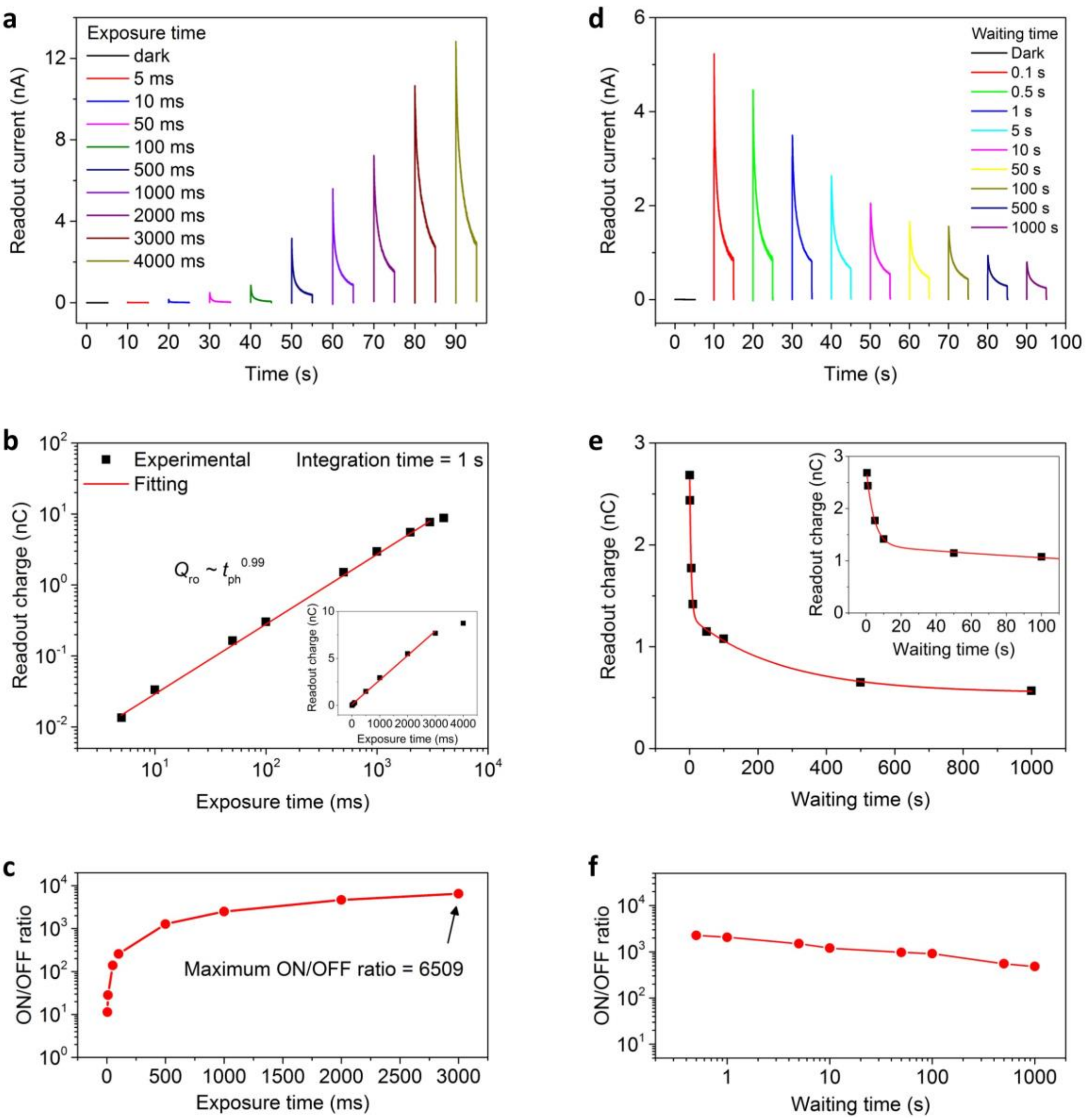

Supplementary Figure 12. Characterization of the MoS_2 optoelectronic device with a 650 nm red laser. a, Readout current as a function of light exposure times. The power of the red laser was 20 nW. **b,** Extracted readout charge as a function of exposure times with an integration time of 1 s (log scale), and power-law function fitting (red). The inset shows that the readout charge (linear scale) saturated above 3000 ms with 7.5 nC, which is almost the same level as the saturated charge level measured by a blue laser (7.1 nC) in Fig. 2b. **c,** The ON/OFF ratio as a function of exposure times. **d,** Readout current as a function of waiting times. **e,** Extracted charge by integrating the readout current for 1 s with increasing waiting

time. The inset is the enlarged graph of the readout charge as a function of waiting times within 100 s. **f,** The ON/OFF ratio and the readout charge for ON- and OFF-state with regard to the waiting times. The ON/OFF ratio was maintained at about 480 even after waiting for 10^3 s.

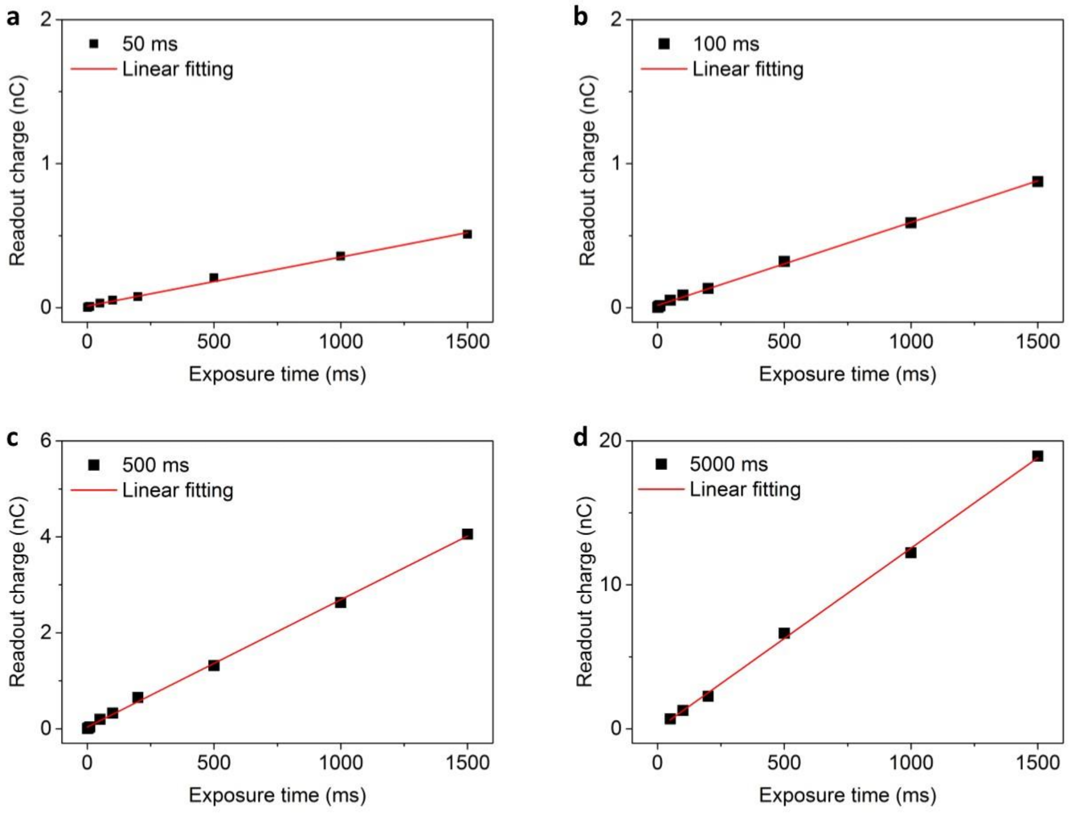

Supplementary Figure 13. Readout charge collected from different integration times. a, Readout charge integrated for 50 ms, **b,** 100 ms, **c,** 500 ms, and **d,** 5000 ms. The light exposure time and the waiting time of the 450 nm laser were 1 s and 0.5 s, respectively. The linear dependence of the readout charge on the light exposure time was also observed at all four different integration times. These results imply that we can control the integration time according to the device requirement.

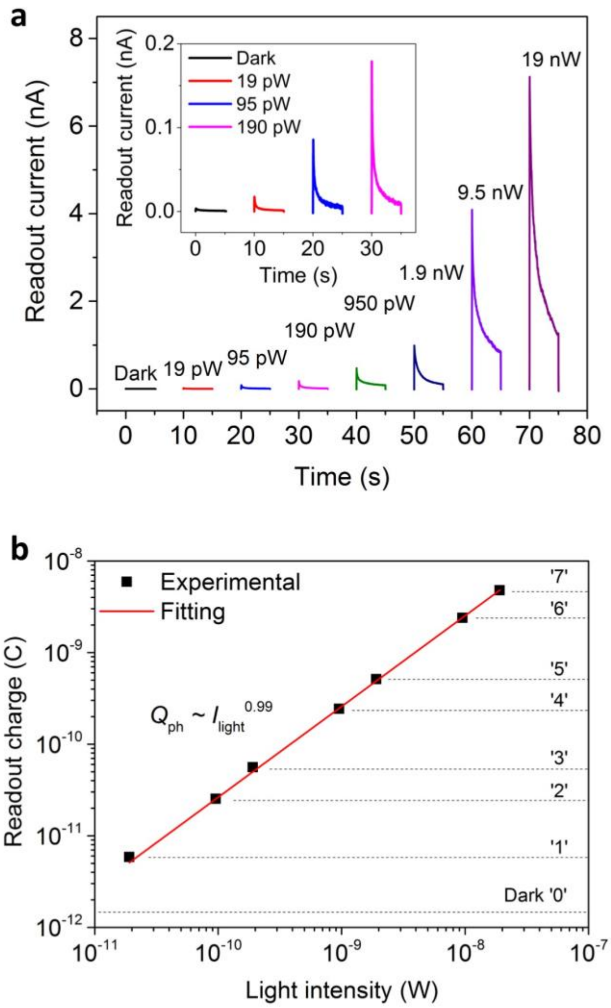

Supplementary Figure 14. Characterization of the optical memory with varying light intensity. a, The readout current as a function of light intensity. The plots were separated with an interval of 10 s, regardless of the time that was measured initially. The light exposure time was 1 s, and the readout bias was applied after a 500 ms waiting time. The inset represents an enlarged view of the readout current for different light intensity from dark conditions up to 190 pW. **b,** The extracted readout charge (log scale) obtained by integrating the readout current for 1 s with increasing light intensity. The fitted line (red), using a power-law function, indicates that the readout charge is linearly dependent on the light intensity. As a result, 8 different states were easily realized with a sufficiently large separation between any two consecutive states.

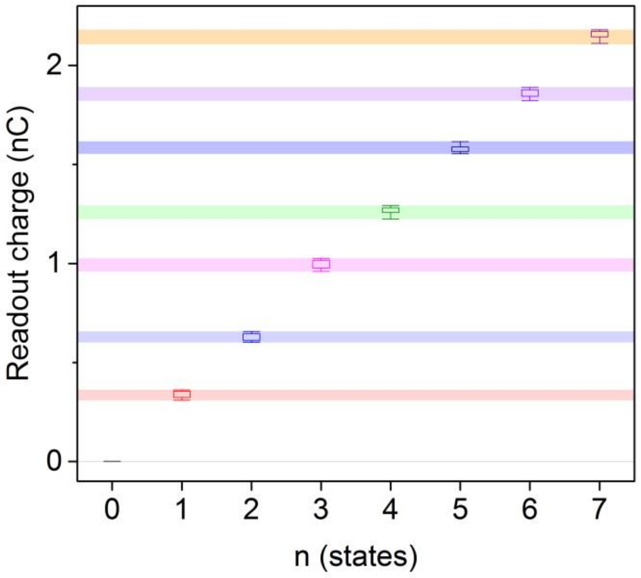

Supplementary Figure 15. Multi-bit operation switching. Box chart of the readout charge for a multi-bit response of the optoelectronic memory device. The readout of each state is demonstrated over 10 cycles, and the confidence interval for each state is color-shaded. The significant differences between the readout charges of any two consecutive states are much larger than the confidence interval of each state (Supplementary Table 1), which shows the possibility of the multi-bit operation.

Supplementary Table 1. Readout charge accuracy in multi-bit operation. Standard deviation for each state calculated in nC and percentage from Figure S15. Standard deviation (%) was calculated by dividing the standard deviation (nC) by the gap in the readout charge between each measured state and the state below.

State	Average readout charge (nC)	Standard deviation (nC)	Standard deviation (%)
**7**	2.1550	0.02030	6.87%
**6**	1.8597	0.01905	6.73%
**5**	1.5766	0.01827	5.89%
**4**	1.2664	0.01885	6.96%
**3**	0.9957	0.02095	5.72%
**2**	0.6295	0.01855	6.35%
**1**	0.3374	0.01656	4.92%
**0**	0.0012	5.90×10^{-5}	–

Supplementary References

1. Lee, C. *et al.* Anomalous lattice vibrations of single- and few-layer MoS_2. *ACS Nano* **4,** 2695–2700 (2010).

2. Li, H. *et al.* From bulk to monolayer MoS_2: Evolution of Raman scattering. *Adv. Funct. Mater.* **22,** 1385–1390 (2012).

3. Ghatak, S., Pal, A. & Ghosh, A. Nature of electronic states in atomically thin MoS_2 field-effect transistors. *ACS Nano* **5,** 7707–7712 (2011).

4. Lee, G-H. *et al.* Flexible and transparent MoS_2 field-effect transistors on hexagonal boron nitride-graphene heterostructures. *ACS Nano* **7,** 7931–7936 (2013).

5. Mak, K. *et al.* Tightly bound trions in monolayer MoS_2. *Nature Mater.* **12,** 207–211 (2012).

6. Zhang, W. *et al.* High-Gain phototransistors based on a CVD MoS_2 monolayer. *Adv. Mat.* **25,** 3456–3461 (2013).

7. Furchi, M., Polyushkin, D., Pospischil, A. & Mueller, T. Mechanisms of photoconductivity in atomically thin MoS_2. *Nano Lett.* **14**, 6165-6170 (2014).

8. Lan, C., Li, C., Yin, Y. & Liu, Y. Large-area synthesis of monolayer WS_2 and its ambient-sensitive photo-detecting performance. *Nanoscale* **7,** 5974–80 (2015).

9. Pradhan, N. *et al.* High photoresponsivity and short photoresponse times in few-layered WSe_2 transistors. *ACS Applied Materials & Interfaces* **7,** 12080–12088 (2015).

10. Abderrahmane, A. *et al.* High photosensitivity few-layered $MoSe_2$ back-gated field-effect phototransistors. *Nanotechnology* **25,** 365202 (2014).
